# Treatment of Hooping Cough by Cochineal

**Published:** 1844-02

**Authors:** 


*Treatment of Hooping-Cough by Cochineal.*—Dr. Cajitan Wacht, of Vienna, has treated nine children affected with hooping-cough, by cochineal, which has been already used for that purpose by English physicians. The medicine has been administered in every stage of the disorder, and its efficacy has been so very instantaneous, so constant, that notwithstanding the small number of cases Mr. Wacht had, he believes himself authorized to announce cochineal a specific in hooping-cough. The following is the mixture he used:

Cochineal, - - - 10 decigrammes.
Sugar, - - - - 32 grammes,
Warm water, - - 192 grammes.

Dissolve. Dose, 3 teaspoonfuls during the 24 hours.

It should not be used after it has been made thirty-six hours, decomposition having then commenced.—*Journ. de. Med.*

*Note by Translator.*—The mixture commonly used in this country is formed of cochineal, ℈j.; salt of tartar, ℈ij.; water, 4 oz.; dose, a teaspoonful. From Mr. Wacht's statement above given, it would appear that its agency in the cure of hooping cough, is due to the cochineal alone.—*American Journal of Pharmacy*, Jan., 1844.

